# Trends and Intensity of Rhinovirus Invasions in Kilifi, Coastal Kenya, Over a 12-Year Period, 2007–2018

**DOI:** 10.1093/ofid/ofab571

**Published:** 2021-11-16

**Authors:** John Mwita Morobe, Everlyn Kamau, Nickson Murunga, Winfred Gatua, Martha M Luka, Clement Lewa, Robinson Cheruiyot, Martin Mutunga, Calleb Odundo, D James Nokes, Charles N Agoti

**Affiliations:** 1 Epidemiology and Demography Department, KEMRI-Wellcome Trust Research, Programme, Centre for Geographic Medicine Research–Coast, Kilifi, Kenya; 2 Nuffield Department of Medicine, University of Oxford, Oxford, United Kingdom; 3 School of Life Sciences and Zeeman Institute for Systems Biology and Infectious Disease Epidemiology Research, University of Warwick, Coventry, United Kingdom; 4 Department of Public Health, Pwani University, Kilifi, Kenya

**Keywords:** coastal Kenya, invasion, long-term surveillance, persistence, rhinovirus

## Abstract

**Background:**

Rhinoviruses (RVs) are ubiquitous pathogens and the principal etiological agents of common cold. Despite the high frequency of RV infections, data describing their long-term epidemiological patterns in a defined population remain limited.

**Methods:**

Here, we analyzed 1070 VP4/VP2 genomic region sequences sampled at Kilifi County Hospital on the Kenya coast. The samples were collected between 2007 and 2018 from hospitalized pediatric patients (<60 months of age) with acute respiratory illness.

**Results:**

Of 7231 children enrolled, RV was detected in 1497 (20.7%) and VP4/VP2 sequences were recovered from 1070 samples (71.5%). A total of 144 different RV types were identified (67 *Rhinovirus A*, 18 *Rhinovirus B*, and 59 *Rhinovirus C*) and at any month, several types co-circulated with alternating predominance. Within types, multiple genetically divergent variants were observed. Ongoing RV infections through time appeared to be a combination of (1) persistent types (observed up to 7 consecutive months), (2) reintroduced genetically distinct variants, and (3) new invasions (average of 8 new types annually).

**Conclusions:**

Sustained RV presence in the Kilifi community is mainly due to frequent invasion by new types and variants rather than continuous transmission of locally established types/variants.

Rhinoviruses (RVs) are a highly prevalent group of viruses and are the principal cause of common cold syndrome in humans globally [1, 2]. RV infections result in a wide range of clinical outcomes spanning from asymptomatic and mild illness in the upper airways to severe illness in the lower airways [3, 4]. The infections occur in all ages, with severe presentation more likely in children under the age of 5 years [5, 6], the elderly [7], and immunocompromised persons [8]. Despite the clinical significance of RV infections, there is little information on the long-term trends and diversity of circulating RV types.

RV belongs to the genus *Enterovirus* of the family Picornaviridae. The viral single-stranded positive sense RNA genome consists of approximately 7200 nucleotides and encodes 4 structural proteins (VP4, VP2, VP3, and VP1) and 7 nonstructural proteins (2A, 2B, 2C, 3A, 3B, 3C, and 3D) [2]. The 3 surface-exposed capsid proteins (VP1, VP2, and VP3) carry the antigenically critical sites [9–11]. The high genetic variability in the VP4/VP2 and VP1 genomic regions of RVs have been instrumental in molecular typing [12, 13] and molecular epidemiological investigations of RV infections [14–16]. Currently, a total of 169 RV types have been described and classified into 3 species: *Rhinovirus A*, *Rhinovirus B*, and *Rhinovirus C* (https://www.picornaviridae.com/sg3_ensavirinae/enterovirus/enterovirus.htm).

RV infections occur year-round in most geographical locations, although peaking in the early autumn and late spring in many temperate countries, and in the rainy season in tropical countries [2, 17]. Unclear seasonality and year-round transmission of RVs have been attributed to lack of intertype cross-protective immunity [18, 19], coupled with the high genetic diversity within the 3 species, each with the ability to spread independently in a population [14, 16, 20].

A recent study in Kilifi County, located in coastal Kenya, that spanned over a 12-month period [14] (December 2015–November 2016) found that multiple RV types co-circulate over varied time periods ranging from 1 to 9 months and, in most cases, each displaying a typical epidemic curve at the local population level; transmission is presumably constrained by the decline in susceptibles to that type within the locality. Type-specific (homologous) immunity has been reported to wane approximately after a 1-year period [21], and individuals who were previously immune to a particular type gradually become susceptible to the type again [21, 22]. Previous studies found that introduction of new RV types or sequential invasion by different genetic variants could be due to declining levels of population immunity as well as viral evolution [23, 24].

These assertions of perpetually changing RV types during year-round RV transmission have not been fully investigated in a longitudinal manner [16]. In this study, we analyzed VP4/VP2 sequences of samples collected from hospitalized children with acute respiratory illness between 2007 and 2018 on the Kenyan coast to evaluate the long-term incidence of the different RV types, their temporal patterns, and intensity of new invasions in a local population.

## MATERIALS AND METHODS

### Study Area and Population

The study was conducted at the Kilifi County Hospital (KCH) as part of long-term surveillance initially aimed at understanding the epidemiology and disease burden of respiratory syncytial virus–associated pneumonia cases [25] and expanded to a range of respiratory viruses from 2007 onward [6, 26–30]. KCH, located on the coast of Kenya, is a referral hospital serving the wider Kilifi County, which has a population of 1453787 and covers an area of approximately 12254 km^2^. Details of study design, participant recruitment, and sampling procedures have been described elsewhere [25, 29]. In brief, upon presentation to the pediatric ward, a detailed medical review was undertaken by the clinician and the decision to admit was made. For this study, children (<60 months of age) admitted to the pediatric ward between January 2007 and December 2018 were eligible if they presented with symptoms of syndromic severe or very severe pneumonia. Clinical definitions include a history of cough or difficulty in breathing for <30 days, which if accompanied by lower chest wall indrawing was defined as severe pneumonia; or if accompanied by any 1 of prostration, coma, or hypoxemia was defined as very severe pneumonia (prostration included the inability to feed or drink, and hypoxemia defined by oxygen saturation [pO_2_] <90%) [25]. Following a written informed consent from the parent or guardian, a nasopharyngeal flocked swab, nasal wash, or combination of nasopharyngeal swab and oropharyngeal swab was collected from each child and transferred into viral transport medium for laboratory screening. Ethical approval for the study protocol was obtained from the Scientific and Ethics Review Unit (SERU number 3443) ethics committee, Kenya Medical Research Institute, Nairobi, Kenya.

## RV SCREENING AND SEQUENCING

Viral RNA was extracted from each sample using QIAamp Viral RNA kit (Qiagen, Valencia, California) and screened for respiratory viruses using a multiplex real-time reverse-transcription PCR (rRT-PCR) (Applied Biosystems, United Kingdom) as described elsewhere [31, 32]. A sample was considered RV positive if the rRT-PCR cycle threshold was <35.0 [30]. A section of VP4/VP2 viral genomic region (~420 nucleotides long) of positive samples was amplified and sequenced as previously described [14]. Consensus sequences were assembled using Sequencher software version 5.4.6 (Gene Codes Corporation, Ann Arbor, Michigan).

## SEQUENCE DATA, RV SPECIES, AND TYPE ASSIGNMENT

VP4/VP2 sequencing and typing were attempted for all the RV-positive samples collected in 2014 and 2016–2018. For the years 2010–2013 and 2015, 100 RV-positive samples were randomly selected for sequencing proportional to the monthly distribution of positive samples (Supplementary Table 1). Previously published VP4/VP2 sequences from Kilifi (January 2007– December 2009) were retrieved from GenBank (n = 271, sequence accession numbers: KY006195–KY006465) and combined with the 799 newly generated VP4/VP2 sequences (January 2010–December 2018, GenBank sequence accession numbers; MW622248–MW623046).

## DEFINITION OF TERMS

We used the term “type” to refer to RV sequences classified by either cross-neutralization or genetic comparisons as distinct as described previously [13]. Based on this approach, sequences were assigned into the same RV type based on >90% nucleotide similarity to RV prototype sequences (also referred to as reference sequences, http://www.picornaviridae.com/sequences/sequences.htm) and phylogenetic clustering with bootstrap support value >70% [13]. Distributions of pairwise genetic distances were assessed for evaluation of intertype and intratype divergence [13]. Intratype “variant” was defined on the basis of a divergence threshold value determined as the least frequent value between the first and second modes in a pairwise nucleotide difference distribution plot. Here we are implicitly assuming that sequences with pairwise nucleotide difference falling into the distribution with the low (first) mode are members of the same phylogenetic clade, whereas those with pairwise nucleotide difference within the second distribution with higher mode are members of different phylogenetic clades. A group of viruses within the first, lower distribution were classified as belonging to the same RV type variant.

The definitions used to describe the temporal occurrence of RV types are summarized as follows:

1. Persistent: Continued detection, in consecutive or nonconsecutive years, of a group of viruses belonging to the same variant of a RV type.
2. Recurrent: Detection of a virus or group of viruses not observed in the preceding years (>1 year) that belong to a different variant of a previously observed RV type.
3. Invasion: Detection of a new RV type not previously locally documented.

## PHYLOGENETIC ANALYSES

Multiple sequence alignments were generated using MAFFT version 7.220 [33] and maximum likelihood phylogenetic trees estimated using IQ-TREE version 1.6.12 [34]. Branch support was assessed by 1000 bootstrap iterations. Temporal signal in the data was examined using TempEst version 1.5.3 [35]. To infer time-scaled phylogenies, Bayesian phylogenetic analyses were undertaken in BEAST version 1.10.4 assuming an uncorrelated log-normal relaxed molecular model [36]. The Markov chain Monte Carlo convergence was assessed in Tracer version 1.5, and maximum clade credibility (MCC) trees were summarized using TreeAnnotator version 1.10.4 with a 10% burn-in. MCC trees were visualized using FigTree version 1.4.4.

## RESULTS

### RV Prevalence in Kilifi, 2007–2018

**Figure 1.**
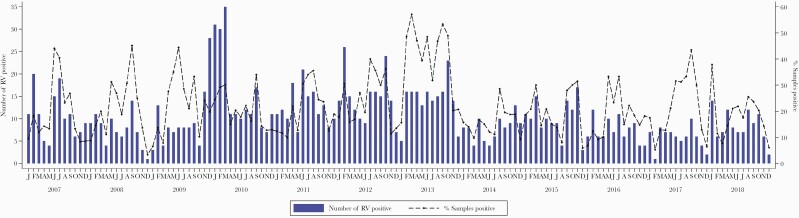
Monthly distribution of rhinovirus (RV) cases identified from surveillance of acute respiratory illness (ARI) in children aged <60 months admitted to the Kilifi County Hospital, Kenya, 2007–2018. Also included on the secondary y-axis are the proportion (% positivity) of the samples from the inpatients with ARI who were RV positive.

Between January 2007 and December 2018, a total of 7231 nasopharyngeal swab samples were collected from children (<60 months of age) admitted with severe or very severe pneumonia in KCH (Supplementary Table 1). RV was detected in 20.7% (1497/7231), with the proportion positive across the years ranging from 15.6% to 38.3% (Supplementary Table 1). The monthly frequency of detection of RV in the study population is shown in Figure 1. RV infections were observed to occur year-round, frequently peaking between the months of May and September each year (Figure 1).

## RV SPECIES AND TYPE ASSIGNMENT

**Figure 2.**
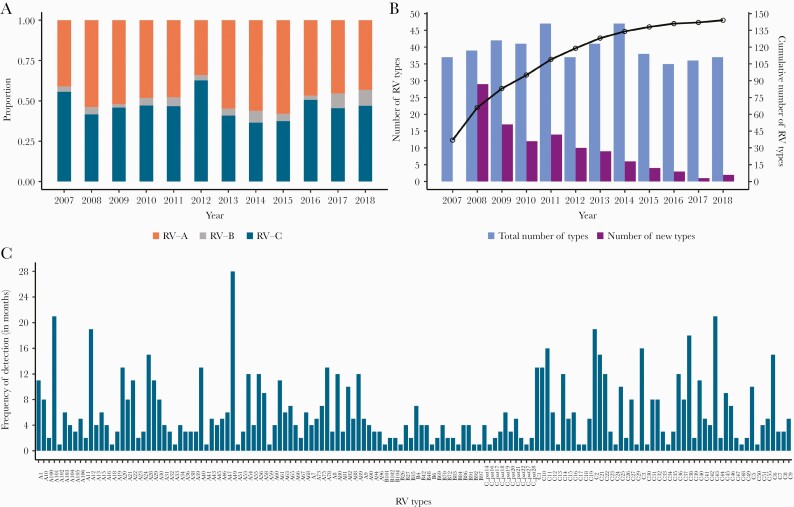
*A*, Annual proportion of rhinovirus (RV) species across the 12-year study period. *B*, Total number (blue bars) and new number (purple bars) of RV types detected annually over the period 2007–2018. Also shown is the cumulative number of the different RV types observed during the study period (black line). *C*, Overall frequency of detection in months or the number of months each RV type was detected. The types are ordered alphabetically.

A total of 1070 (71.5%) VP4/VP2 sequences (~420 nucleotides, some previously reported [30]) were available for this analysis. Of these, 520 (48.6%) sequences were classified as *Rhinovirus A* comprising 67 distinct types; 52 (4.7%) sequences were *Rhinovirus B* comprising 18 types; and 498 (46.5%) were *Rhinovirus C* comprising 59 types. *Rhinovirus A* and *Rhinovirus C* were more frequently detected, whereas *Rhinovirus B* infections were low in number and sporadic (Figure 2A). The most commonly detected types were RV-A49 (n = 39), C2 (n = 29), C38 (n = 26), C11 (n = 26), A101 (n = 24), A12 (n = 23), C6 (n = 22), C21 (n = 21), C3 (n = 20), and A78 (n = 19) (Table 1). Twenty-four sequences could not be assigned to known RV types based on the criterion proposed by McIntyre et al [13] due to these sequences having *p*-distance of >10.5% with respect to their closest reference sequences (Supplementary Table 2). Other enteroviruses were also detected on sequencing the rRT-PCR RV detections: enterovirus D68 (EV-D68) (n = 5), coxsackievirus B3 (CVB3) (n = 1), coxsackievirus B2 (CVB2) (n = 1), and echovirus 19 (E19) (n = 1).

**Table 1.** Number of Different Rhinovirus Types Identified in Kilifi, Kenya, 2007–2018

Type of Rhinovirus (No.)															
RV-A															
A49	A101	A12	A78	A56	A89	A20	A28	A40	A54	A1	A22	A61	A80	A29	A30
(39)	(24)	(23)	(19)	(18)	(18)	(17)	(16)	(16)	(14)	(13)	(13)	(13)	(12)	(11)	(10)
A58	A63	A82	A21	A75	A10	A47	A65	A106	A68	A103	A15	A43	A81	A88	A9
(10)	(10)	(10)	(9)	(9)	(8)	(8)	(8)	(7)	(7)	(6)	(6)	(6)	(6)	(6)	(6)
A16	A31	A46	A60	A66	A73	A104	A105	A13	A34	A36	A45	A55	A7	A90	A19
(5)	(5)	(5)	(5)	(5)	(5)	(4)	(4)	(4)	(4)	(4)	(4)	(4)	(4)	(4)	(3)
A24	A32	A38	A39	A53	A8	A94	A96	A100	A102	A11	A23	A67	A18	A33	A41
(3)	(3)	(3)	(3)	(3)	(3)	(3)	(3)	(2)	(2)	(2)	(2)	(2)	(1)	(1)	(1)
A51	A59	A (untyped)													
(1)	(1)	(13)													
RV-B															
B4	B70	B27	B42	B48	B86	B91	B104	B69	B102	B35	B72	B83	B101	B26	B6
(7)	(5)	(4)	(4)	(4)	(4)	(4)	(3)	(3)	(2)	(2)	(2)	(2)	(1)	(1)	(1)
B84	B92	B97													
(1)	(1)	(1)													
RV-C															
C2	C38	C11	C43	C6	C21	C3	C10	C1	C14	C22	C40	C5	C27	C36	C25
(29)	(26)	(26)	(26)	(22)	(21)	(20)	(18)	(17)	(16)	(16)	(16)	(16)	(15)	(13)	(12)
C45	C37	C31	C32	C9	Cpat19	C46	Cpat18	C12	C16	C55	C15	C19	C41	C51	Cpat14
(12)	(11)	(10)	(10)	(10)	(9)	(8)	(8)	(7)	(7)	(6)	(5)	(5)	(5)	(5)	(5)
Cpat21	C42	Cpat20	C23	C33	C35	C39	C7	C8	C26	C44	C47	C49	Cpat17	Cpat22	Cpat28
(5)	(4)	(4)	(3)	(3)	(3)	(3)	(3)	(3)	(2)	(2)	(2)	(2)	(2)	(2)	(2)
C13	C17	C18	C24	C29	C30	C34	C48	C50	Cpat16	Cpat27	C (untyped)				
(1)	(1)	(1)	(1)	(1)	(1)	(1)	(1)	(1)	(1)	(1)	(11)				

Abbreviation: RV, rhinovirus.

## TEMPORAL TRENDS OF RV TYPES IN KILIFI

We detected on average, 39 RV types annually (range, 35–47), a mean of 8 (range, 1–29) of which were new RV types identified for the first time in the population each year from 2008 as other previously detected types disappeared (Figure 2B). The cumulative number of new RV types detected annually increased rapidly since the beginning of the surveillance period and then saturated after approximately 9 years (Figure 2B). RV types commonly co-circulated and with varying frequency in the 12-year period (Figure 2C, Supplementary File 1). Several types were present at high prevalence whereas others occurred once or sporadically. Some types circulated consecutively for months; for example, RV-A56 was detected in 7 consecutive months (May–November 2007); RV-C11 was present for 6 consecutive months (February–July 2016); and RV-C38, A40, and C2 types circulated consecutively for 5 months (November 2009 to March 2010, April–August 2016, and May–September 2010, respectively) (Supplementary File 1).

**Figure 3.**
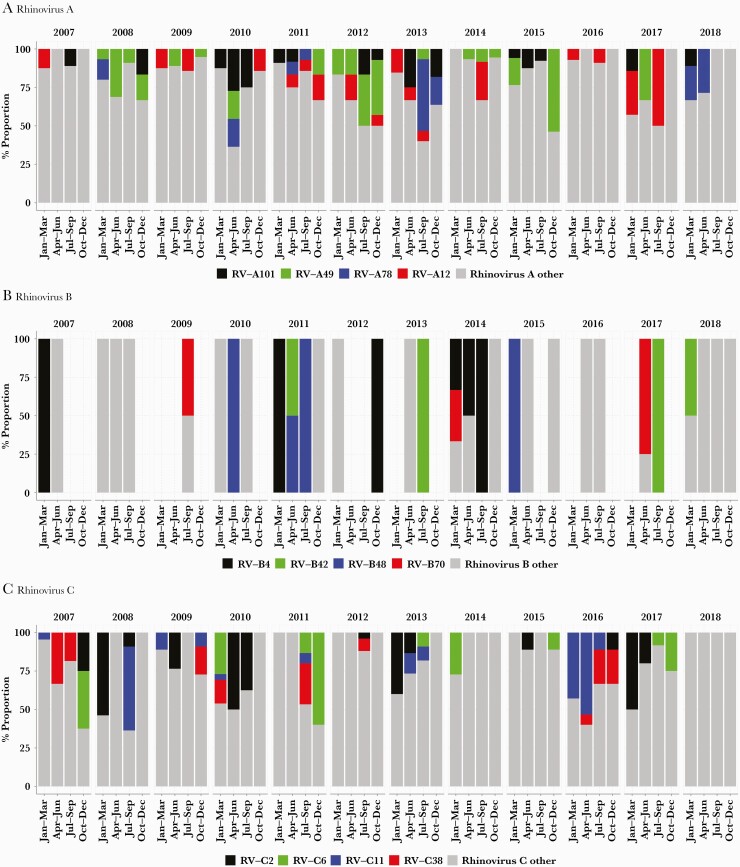
Quarterly proportions of rhinovirus (RV) types detected organized at the species level; shown here are the temporal trends of the 5 most prevalent types per species while the rest are indicated as “other”. *A*, Quarterly proportion of RV-A types. *B*, Quarterly proportion of RV-B types. *C*, Quarterly proportion of RV-C types.

Additionally, several types recurred after considerable periods of absence. For example, RV-A12, first seen in February 2007, was not detected again until February 2009, 23 months later, whereas C38 viruses were detected 4 years apart between 2012 and 2016 (Figure 3, Supplementary File 1). Temporally, several RV types exhibited synchronized co-circulation and recurrence, for example: (1) RV-C1, C11, C2, C38, C22, and C21; (2) RV-A75, A89, A12, A28, A96, A106, A80, and A10; (3) RV-A90, A55, A61, A45, A54, and A60; (4) RV-C14, C41, C45, C10, C16, C25, C32, and C47.

## GENETIC DIVERSITY OF RV TYPES IN KILIFI

**Figure 4.**
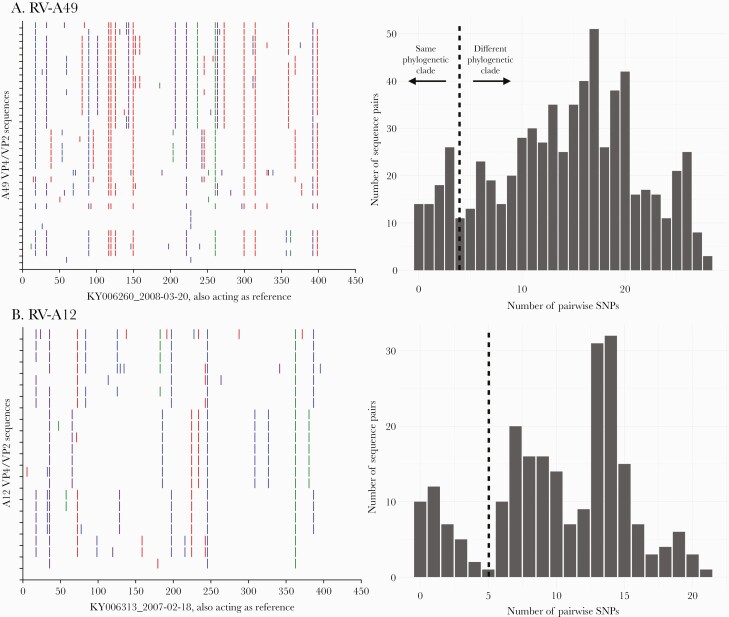
*A*, Nucleotide variability across the sequenced VP4/VP2 region for rhinovirus types RV-A49 and RV-C2. For each type, the viruses were compared to the earliest sampled sequence. Vertical colored bars show the nucleotide differences: Red is a change to T, orange is a change to A, purple is a change to C, and blue is a change to G. *B*, Distribution of pairwise nucleotide difference for the VP4/VP2 region of types RV-A49 and C2. Abbreviations: RV, rhinovirus; SNP, single-nucleotide polymorphism.

The nucleotide sequence identity among *Rhinovirus A*, *Rhinovirus B*, and *Rhinovirus C* viruses was determined as 57.3%–100%, 66.0%–100%, and 45.1%–100%, respectively, and 59.8%–100%, 79.9%–100% and 53.3%–100% at the amino acid level, respectively. Intratype nucleotide variation was observed in the VP4/VP2 region of viruses sampled over the 12-year study period (Figure 4A, Supplementary Figure 1). Nonetheless, the substitutions were mostly synonymous, that is, not amino acid changing. The distribution of pairwise nucleotide distances showed multimodal peaks suggesting circulation of distinct variants within individual RV types (Figure 4B, Supplementary Figure 2). These observations were congruent with multiple within-type phylogenetic clusters.

**Figure 5.**
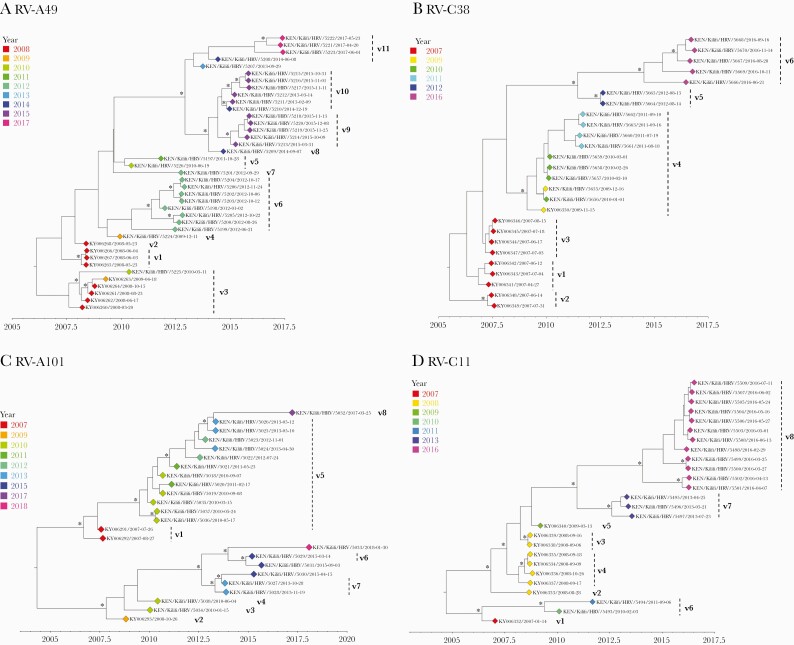
Bayesian phylogenetic trees showing the VP4/VP2 region of the rhinovirus (RV) types A49, C2, C38, and C11. Variant names are next to the phylogenetic clusters, eg, v1 representing variant 1 for a specific type. Node support is indicated by (∗) for posterior probabilities >0.9.

Several RV types were characterized by genetically distinct temporal clusters, for example, RV-A49, C38, and A101 (Figure 5). RV-A49 was detected as 11 distinct variants circulating at different periods, 3 of which occurred as singletons (single sequences), suggesting undersampled genetic diversity (Figure 5, Table 2). Multiple genetic variants of type RV-C6 co-circulated during 2010–2011 (Supplementary Figure 3, Table 2), which likely indicates separate virus introductions into the Kilifi population. Several RV types had variants that contained sequences from multiple years indicating variant persistence over an extended period or repeated reintroductions, for example, RV-A101 variant 5 comprised of viruses observed from 2010 to 2013 (Figure 5, Table 2).

**Table 2.** Number of Variants for the 10 Most Prevalent Rhinovirus Types Identified in Kilifi, Kenya, 2007–2018

Rhinovirus Type	No. of Sequences	No. of Variants, Singletons	Period (Year)
A12	23	9, 5	v1 (2007), v2 (2007), v3 (2009), v4 (2009–2011), v5 (2014), v6 (2014), v7 (2016), v8 (2016), v9 (2017)
A78	19	7, 3	v1 (2008), v2 (2010–2011), v3 (2011), v4 (2013), v5 (2013), v6 (2018), v7 (2018)
C2	29	12, 5	v1 (2007–2008), v2 (2008), v3 (2008), v4 (2009), v5 (2009), v6 (2010), v7 (2010), v8 (2012), v9 (2013), v10 (2015), v11 (2016–2017), v12 (2017)
C11	25	8, 3	v1 (2007), v2 (2008), v3 (2008), v4 (2008), v5 (2009), v6 (2010–2011), v7 (2013), v8 (2016)
C21	21	6, 2	v1 (2007), v2 (2007–2012), v3 (2010), v4 (2012), v5 (2015), v6 (2018)
C38	26	6, 0	v1 (2007), v2 (2007), v3 (2007), v4 (2009–2011), v5 (2012), v6 (2016)
C3	20	7, 2	v1 (2009), v2 (2010–201), v3 (2011), v4 (2013), v5 (2015), v6 (2017), v7 (2018)
A49	39	11, 4	v1 (2008), v2 (2008), v3 (2008–2010), v4 (2009), v5 (2010–2011), v6 (2012), v7 (2012), v8 (2014), v9 (2015), v10 (2014–2015), v11 (2013–2014, 2017)
A101	25	9, 5	v1 (2007), v2 (2008), v3 (2010), v4 (2010), v5 (2010–2013), v6 (2015), v7 (2013–2015), v8 (2017), v9 (2018)
C6	22	11, 4	v1 (2007), v2 (2010–2011), v3 (2010), v4 (2010), v5 (2010), v6 (2011), v7 (2011), v8 (2011), v9 (2013–2014), v10 (2015), v11 (2017)

## DISCUSSION

We describe the long-term pattern of co-circulation, persistence, and invasion of RV types in hospitalized children (<60 months old) with pneumonia in Kilifi, coastal Kenya, over a 12-year period (2007–2018). Consistent with other studies, RV was ubiquitous and multiple types co-circulated even within a single month [14, 16, 20]. Among the RV cases detected, *Rhinovirus B* was least frequently detected. It is not clear why *Rhinovirus B* is less diverse and each type within it was on average less frequent. The observed annual proportions of RV species in Kilifi are consistent with recent similar epidemiological studies in Brazil, Nigeria, and Cameroon [37–39]. Although children <5 years of age are not a comprehensive representative of the community, this demographic gives insight into the pattern of RV transmission since RV burden is highest in children <5 years of age [14]. RV detection rates decrease with increasing age as adults have had multiple and widespread exposures to RV types [14]. Other social groups are vital in RV transmission [6, 40, 41] and it would be useful to evaluate RV transmission patterns and prevalence within these groups.

The majority (99%) of our sequences were within the proposed divergence thresholds for RV typing and classification using the VP4/VP2 region (10.5% for *Rhinovirus A*, 9.5% for *Rhinovirus B*, and 10.5% for *Rhinovirus C*) [13]. This exemplifies significant sequence conservation in the VP4/VP2 region within a type allowing robust genotypic assignment. However, 24 sequences did not fit the classification system for VP4/VP2 region and require whole genome sequencing to check for variation in the VP1 region and determine if they correspond to new types [13]. Detection of other enteroviruses reflects PCR cross-reactivity due to nucleotide conservation at the 5ʹ-untranslated target region [42, 43]. EV-D68, CVB3, CVB2, and E19 have been associated with respiratory disease or detected in respiratory samples [44, 45].

The frequent invasions of new types could be explained by lack of preexisting immune memory or weak heterotypic immunity [46]. The number of new types decreased over time, levelling off in 2016, perhaps indicating the period a population takes to experience the maximum number of RV types. Recurrence of RV types could be promoted by antigenic variation on the other surface proteins (VP2, VP1, and VP3) allowing infection where prior exposure confers incomplete or short-lived immunity to future genetic variants. Recurrence, particularly where the recurring strains were genetically identical to older strains, may also be observed in a population not previously exposed to a RV type. Some RV types occurred sporadically and could be associated with mild disease or asymptomatic infections or have reduced transmission rates probably suppressed by preexisting immunity [47].

For some RV types, the sequenced VP4/VP2 region remained conserved after periods of quiescence, which probably ensures strain survival by maintaining low-level genetic variation. In a linear strain space, strains interact via cross-immunity to nearby strains with shared epitopes, and this interaction tails off with genetic distance [48]. Yet, the VP4/VP2 region might not be primarily antigenic [49], and genetic changes could have occurred at immunogenic sites located in other capsid proteins (VP1 or VP3). Genome-wide sequence data would therefore be useful to confirm strain conservation and maintenance. The evident intratype genetic diversity with differential temporal distribution could suggest sequential virus introductions or diversification of locally circulating variants [50].

This study had 2 limitations. First, in some years (2010–2013 and 2015) we only sequenced a proportion of the positive cases (Supplementary Table 1), which might underestimate the circulating RV diversity. Samples selected were prioritized based on viral load and monthly distribution. Second, we only sequenced the VP4/VP2 coding region, but more reliable phylogenetic relationships would be defined from full-length genome analysis [51].

In conclusion, this study describes the nature of RV infections in hospitalized children <60 months old and enhances our understanding on RV transmission dynamics in a community. RV dynamics in Kilifi during 2007–2018 were characterized by repeated invasions by heterogeneous types rather than long-term continuity of the same RV types and continuous diversification of circulating variants. Improved understanding on the RV types circulating in a community may support better guidance of future therapeutic interventions in clinical practice. The high diversity and rates of invasion of RV as observed in this study, even within a short duration (week or month), underpins the application of molecular typing for surveillance and understanding virus epidemiological dynamics.

## Supplementary Data

Supplementary materials are available at *Open Forum Infectious Diseases* online. Consisting of data provided by the authors to benefit the reader, the posted materials are not copyedited and are the sole responsibility of the authors, so questions or comments should be addressed to the corresponding author.

ofab571_suppl_Supplementary_Figure_S1

ofab571_suppl_Supplementary_Figure_S2

ofab571_suppl_Supplementary_Figure_S3

ofab571_suppl_Supplementary_Table_S1

ofab571_suppl_Supplementary_Table_S2

ofab571_suppl_Supplementary_File_S1

ofab571_suppl_Supplementary_Materials

## Notes

***Author contributions.*** J. M. M., E. K., C. A., and D. J. N. designed the study. J. M. M., C. L., R. C., M. M., and C. O. performed rhinovirus screening and sequencing. J. M. M., E. K., M. M. L., and N. M. curated and analyzed data. J. M. M., E. K., C. A., and D. J. N. wrote the manuscript. All authors have read and approved the final manuscript draft.

***Acknowledgments.*** We thank the study participants for providing the study samples. We also thank all members of the Virus Epidemiology and Control (VEC) Research Group in Kilifi who were involved at various stages of this study. We also thank the parents and guardians of the children for agreeing to participate in this study.

***Data availability.*** All data generated and analysis script for this manuscript are available from the Virus Epidemiology and Control, Kenya Medical Research Institute (KEMRI)–Wellcome Trust Research Programme, data server: https://doi.org/10.7910/DVN/OL399P.

***Disclaimer.*** The funder had no role in other aspects of the study including its design, data collection, data analysis, data interpretation, or writing of this manuscript. This was submitted for publication with permission from Director of KEMRI.

***Financial support.*** This study was supported by the Wellcome Trust, United Kingdom (grant number 102975).

***Potential conflicts of interest.*** All authors: No reported conflicts of interest.

## References

[1] Ouédraogo S , Traoré B, Nene Bi ZA, et al. Viral etiology of respiratory tract infections in children at the pediatric hospital in Ouagadougou (Burkina Faso). PLoS One 2014; 9:e110435. 25360527 10.1371/journal.pone.0110435 PMC4215928

[2] Jacobs SE , Lamson DM, St George K, Walsh TJ. Human rhinoviruses. Clin Microbiol Rev 2013; 26:135–62. 23297263 10.1128/CMR.00077-12 PMC3553670

[3] Fry AM , Lu X, Olsen SJ, et al. Human rhinovirus infections in rural Thailand: epidemiological evidence for rhinovirus as both pathogen and bystander. PLoS One 2011; 6:e17780. 21479259 10.1371/journal.pone.0017780 PMC3066183

[4] van Benten I , Koopman L, Niesters B, et al. Predominance of rhinovirus in the nose of symptomatic and asymptomatic infants. Pediatr Allergy Immunol 2003; 14:363–70. 14641606 10.1034/j.1399-3038.2003.00064.x PMC7168036

[5] Miller EK , Lu X, Erdman DD, et al; New Vaccine Surveillance Network. Rhinovirus-associated hospitalizations in young children. J Infect Dis 2007; 195:773–81. 17299706 10.1086/511821 PMC7109786

[6] Luka MM , Kamau E, Adema I, et al. Molecular epidemiology of human rhinovirus from 1-year surveillance within a school setting in rural coastal Kenya. Open Forum Infect Dis 2020; 7:ofaa385. 33094115 10.1093/ofid/ofaa385 PMC7568438

[7] Hung IFN , Zhang AJ, To KKW, et al. Unexpectedly higher morbidity and mortality of hospitalized elderly patients associated with rhinovirus compared with influenza virus respiratory tract infection. Int J Mol Sci 2017; 18:259. 10.3390/ijms18020259 PMC5343795 28134768

[8] Piralla A , Zecca M, Comoli P, et al. Persistent rhinovirus infection in pediatric hematopoietic stem cell transplant recipients with impaired cellular immunity. J Clin Virol 2015; 67:38–42. 25959156 10.1016/j.jcv.2015.03.022 PMC7172262

[9] Sherry B , Rueckert R. Evidence for at least two dominant neutralization antigens on human rhinovirus 14. J Virol 1985; 53:137–43. 2981332 10.1128/jvi.53.1.137-143.1985 PMC254989

[10] Sherry B , Mosser AG, Colonno RJ, Rueckert RR. Use of monoclonal antibodies to identify four neutralization immunogens on a common cold picornavirus, human rhinovirus 14. J Virol 1986; 57:246–57. 2416951 10.1128/jvi.57.1.246-257.1986 PMC252721

[11] Stepanova E , Isakova-Sivak I, Rudenko L. Overview of human rhinovirus immunogenic epitopes for rational vaccine design. Expert Rev Vaccines 2019; 18:877–80. 31416365 10.1080/14760584.2019.1657014

[12] Simmonds P , McIntyre C, Savolainen-Kopra C, et al. Proposals for the classification of human rhinovirus species C into genotypically assigned types. J Gen Virol 2010; 91:2409–19. 20610666 10.1099/vir.0.023994-0

[13] McIntyre CL , Knowles NJ, Simmonds P. Proposals for the classification of human rhinovirus species A, B and C into genotypically assigned types. J Gen Virol 2013; 94:1791–806. 23677786 10.1099/vir.0.053686-0 PMC3749525

[14] Morobe JM , Nyiro JU, Brand S, et al. Human rhinovirus spatial-temporal epidemiology in rural coastal Kenya, 2015-2016, observed through outpatient surveillance. Wellcome Open Res 2019; 3:128. 30483602 10.12688/wellcomeopenres.14836.1 PMC6234744

[15] Arakawa M , Okamoto-Nakagawa R, Toda S, et al. Molecular epidemiological study of human rhinovirus species A, B and C from patients with acute respiratory illnesses in Japan. J Med Microbiol 2012; 61:410–9. 22016561 10.1099/jmm.0.035006-0

[16] van der Linden L , Bruning AHL, Thomas XV, et al. A molecular epidemiological perspective of rhinovirus types circulating in Amsterdam from 2007 to 2012. Clin Microbiol Infect 2016; 22:1002.e9–14. 10.1016/j.cmi.2016.08.007 PMC7129042 27554204

[17] Garcia J , Espejo V, Nelson M, et al. Human rhinoviruses and enteroviruses in influenza-like illness in Latin America. Virol J 2013; 10:305. 24119298 10.1186/1743-422X-10-305 PMC3854537

[18] Glanville N , Johnston SL. Challenges in developing a cross-serotype rhinovirus vaccine. Curr Opin Virol 2015; 11:83–8. 25829255 10.1016/j.coviro.2015.03.004

[19] Glanville N , McLean GR, Guy B, et al. Cross-serotype immunity induced by immunization with a conserved rhinovirus capsid protein. PLoS Pathog 2013; 9:e1003669. 24086140 10.1371/journal.ppat.1003669 PMC3784482

[20] Sansone M , Andersson M, Brittain-Long R, et al. Rhinovirus infections in western Sweden: a four-year molecular epidemiology study comparing local and globally appearing types. Eur J Clin Microbiol Infect Dis 2013; 32:947–54. 23435753 10.1007/s10096-013-1832-x PMC7087832

[21] Barclay WS , al-Nakib W, Higgins PG, Tyrrell DA. The time course of the humoral immune response to rhinovirus infection. Epidemiol Infect 1989; 103:659–69. 2558033 10.1017/s095026880003106x PMC2249538

[22] Barclay WS , Callow KA, Sergeant M, al-Nakib W. Evaluation of an enzyme-linked immunosorbent assay that measures rhinovirus-specific antibodies in human sera and nasal secretions. J Med Virol 1988; 25:475–82. 2844987 10.1002/jmv.1890250411 PMC7166658

[23] Jartti T , Lee WM, Pappas T, et al. Serial viral infections in infants with recurrent respiratory illnesses. Eur Respir J 2008; 32:314–20. 18448489 10.1183/09031936.00161907 PMC2843696

[24] van der Zalm MM , Wilbrink B, van Ewijk BE, et al. Highly frequent infections with human rhinovirus in healthy young children: a longitudinal cohort study. J Clin Virol 2011; 52:317–20. 21982210 10.1016/j.jcv.2011.09.003

[25] Nokes DJ , Ngama M, Bett A, et al. Incidence and severity of respiratory syncytial virus pneumonia in rural Kenyan children identified through hospital surveillance. Clin Infect Dis 2009; 49:1341–9. 19788358 10.1086/606055 PMC2762474

[26] Berkley JA , Munywoki P, Ngama M, et al. Viral etiology of severe pneumonia among Kenyan infants and children. JAMA 2010; 303:2051–7. 20501927 10.1001/jama.2010.675 PMC2968755

[27] Hammitt LL , Kazungu S, Morpeth SC, et al. A preliminary study of pneumonia etiology among hospitalized children in Kenya. Clin Infect Dis 2012; 54(Suppl 2):S190–9. 22403235 10.1093/cid/cir1071 PMC3297554

[28] Oketch JW , Kamau E, Otieno GP, et al. Human metapneumovirus prevalence and patterns of subgroup persistence identified through surveillance of pediatric pneumonia hospital admissions in coastal Kenya, 2007-2016. BMC Infect Dis 2019; 19:757. 31470805 10.1186/s12879-019-4381-9 PMC6716807

[29] Otieno GP , Murunga N, Agoti CN, et al. Surveillance of endemic human coronaviruses (HCoV-NL63, OC43 and 229E) associated with childhood pneumonia in Kilifi, Kenya. Wellcome Open Res 2020; 5:150. 32995556 10.12688/wellcomeopenres.16037.1 PMC7512035

[30] Onyango CO , Welch SR, Munywoki PK, et al. Molecular epidemiology of human rhinovirus infections in Kilifi, coastal Kenya. J Med Virol 2012; 84:823–31. 22431032 10.1002/jmv.23251 PMC3500870

[31] Gunson RN , Collins TC, Carman WF. Real-time RT-PCR detection of 12 respiratory viral infections in four triplex reactions. J Clin Virol 2005; 33:341–4. 15927526 10.1016/j.jcv.2004.11.025 PMC7108440

[32] Hammitt LL , Kazungu S, Welch S, et al. Added value of an oropharyngeal swab in detection of viruses in children hospitalized with lower respiratory tract infection. J Clin Microbiol 2011; 49:2318–20. 21490188 10.1128/JCM.02605-10 PMC3122752

[33] Katoh K , Standley DM. MAFFT multiple sequence alignment software version 7: improvements in performance and usability. Mol Biol Evol 2013; 30:772–80. 23329690 10.1093/molbev/mst010 PMC3603318

[34] Nguyen LT , Schmidt HA, von Haeseler A, Minh BQ. IQ-TREE: a fast and effective stochastic algorithm for estimating maximum-likelihood phylogenies. Mol Biol Evol 2015; 32:268–74. 25371430 10.1093/molbev/msu300 PMC4271533

[35] Rambaut A , Lam TT, Max Carvalho L, Pybus OG. Exploring the temporal structure of heterochronous sequences using TempEst (formerly Path-O-Gen). Virus Evol 2016; 2:vew007. 27774300 10.1093/ve/vew007 PMC4989882

[36] Li WL , Drummond AJ. Model averaging and Bayes factor calculation of relaxed molecular clocks in Bayesian phylogenetics. Mol Biol Evol 2012; 29:751–61. 21940644 10.1093/molbev/msr232 PMC3258040

[37] Oluwasemowo OO , Nejo YT, Abokede JO, et al. Genotypes of rhinovirus detected among children in two communities of south-west Nigeria. Virus Genes 2021; 57:276–9. 33988838 10.1007/s11262-021-01841-0

[38] Kenmoe S , Sadeuh-Mba SA, Vernet MA, et al. Molecular epidemiology of enteroviruses and rhinoviruses in patients with acute respiratory infections in Yaounde, Cameroon. Influenza Other Respir Viruses 2021; 15:641–50. 33694322 10.1111/irv.12851 PMC8404047

[39] da Costa Souza L , Bello EJM, Dos Santos EM, Nagata T. Molecular and clinical characteristics related to rhinovirus infection in Brasília, Brazil. Braz J Microbiol 2021; 52:289–98. 33410102 10.1007/s42770-020-00411-0 PMC7787651

[40] Peltola V , Waris M, Osterback R, et al. Rhinovirus transmission within families with children: incidence of symptomatic and asymptomatic infections. J Infect Dis 2008; 197:382–9. 18248302 10.1086/525542

[41] Kamau E , Onyango CO, Otieno GP, et al. An intensive, active surveillance reveals continuous invasion and high diversity of rhinovirus in households. J Infect Dis 2019; 219:1049–57. 30576538 10.1093/infdis/jiy621 PMC6420174

[42] Tapparel C , Cordey S, Van Belle S, et al. New molecular detection tools adapted to emerging rhinoviruses and enteroviruses. J Clin Microbiol 2009; 47:1742–9. 19339471 10.1128/JCM.02339-08 PMC2691104

[43] Oberste MS , Nix WA, Maher K, Pallansch MA. Improved molecular identification of enteroviruses by RT-PCR and amplicon sequencing. J Clin Virol 2003; 26:375–7. 12637088 10.1016/s1386-6532(03)00004-0

[44] Royston L , Tapparel C. Rhinoviruses and respiratory enteroviruses: not as simple as ABC. Viruses 2016; 8:16. 10.3390/v8010016 PMC4728576 26761027

[45] Xiang Z , Wang J. Enterovirus D68 and human respiratory infections. Semin Respir Crit Care Med 2016; 37:578–85. 27486738 10.1055/s-0036-1584795 PMC7171721

[46] Rathe JA , Liu X, Tallon LJ, et al. Full-genome sequence and analysis of a novel human rhinovirus strain within a divergent HRV-A clade. Arch Virol 2010; 155:83–7. 19936613 10.1007/s00705-009-0549-8 PMC2910715

[47] Hamilton W , Weiss RA, Wain–Hobson S, et al. Infectious disease dynamics: what characterizes a successful invader? Philos Trans R Soc London Ser B Biol Sci 2001; 356:901–10. 11405937 10.1098/rstb.2001.0866 PMC1088483

[48] Gog JR , Grenfell BT. Dynamics and selection of many-strain pathogens. Proc Natl Acad Sci U S A 2002; 99:17209–14. 12481034 10.1073/pnas.252512799 PMC139294

[49] Patel NR , Demenczuk TM, Watanyar A, et al. VP1 sequencing of all human rhinovirus serotypes: insights into genus phylogeny and susceptibility to antiviral capsid-binding compounds. J Virol 2004; 78:3663–74. 15016887 10.1128/JVI.78.7.3663-3674.2004 PMC371056

[50] Agoti CN , Otieno JR, Ngama M, et al. Successive respiratory syncytial virus epidemics in local populations arise from multiple variant introductions, providing insights into virus persistence. J Virol 2015; 89:11630–42. 26355091 10.1128/JVI.01972-15 PMC4645665

[51] Luka MM , Kamau E, de Laurent ZR, et al. Whole genome sequencing of two human rhinovirus A types (A101 and A15) detected in Kenya, 2016-2018. Wellcome Open Res 2021; 6:178. 34522789 10.12688/wellcomeopenres.16911.1 PMC8408540

