# Supplementary figures and images for: Trends and Intensity of Rhinovirus Invasions in Kilifi, Coastal Kenya, Over a 12-Year Period, 2007–2018
Source: Open Forum Infect Dis. 2021 Nov 16;8(12):ofab571. doi: 10.1093/ofid/ofab571 (PMC8694214; doi:10.1093/ofid/ofab571)

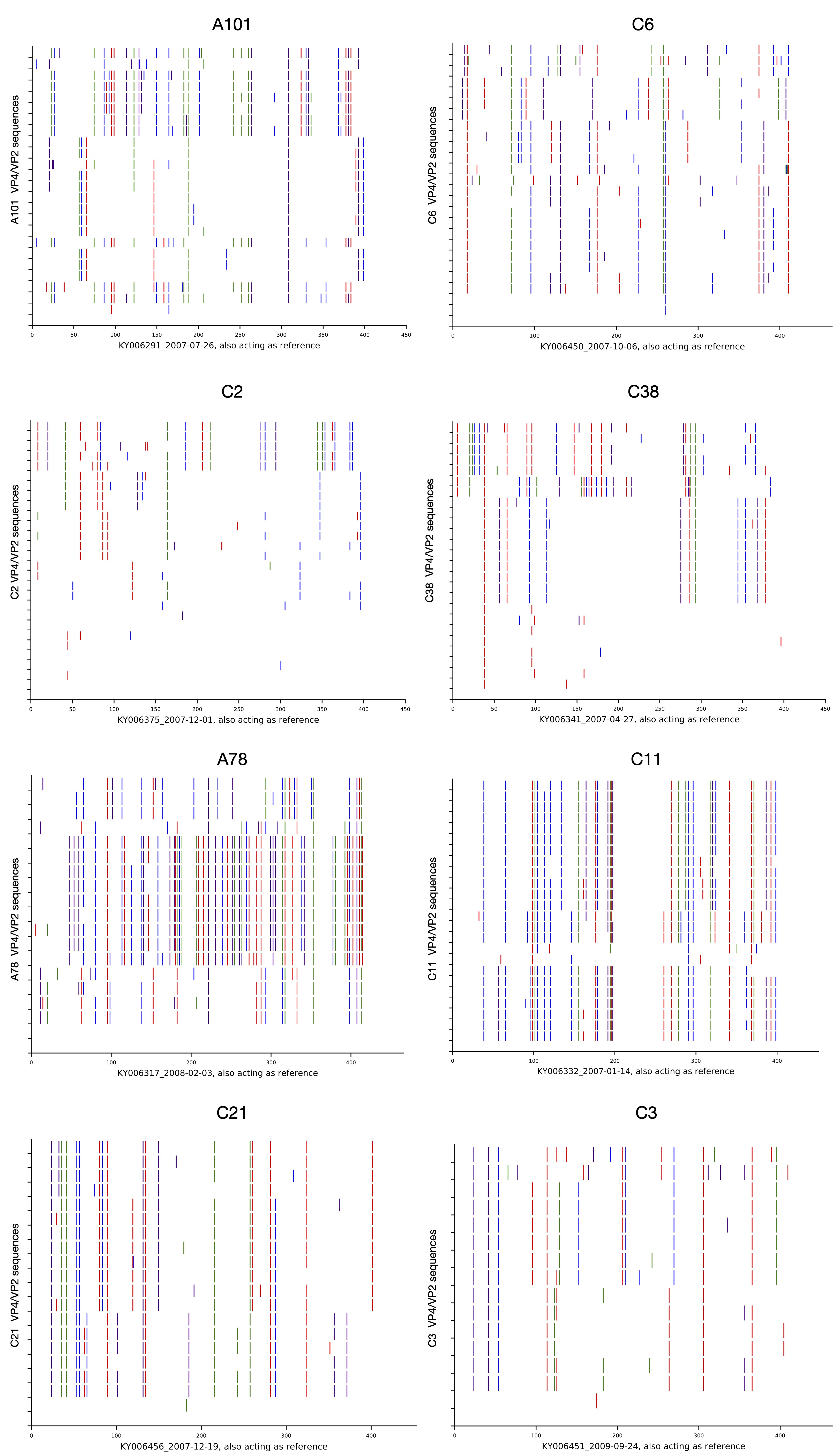

Supplement: ofab571_suppl_Supplementary_Figure_S1 [file ofab571_suppl_supplementary_figure_s1.jpeg]

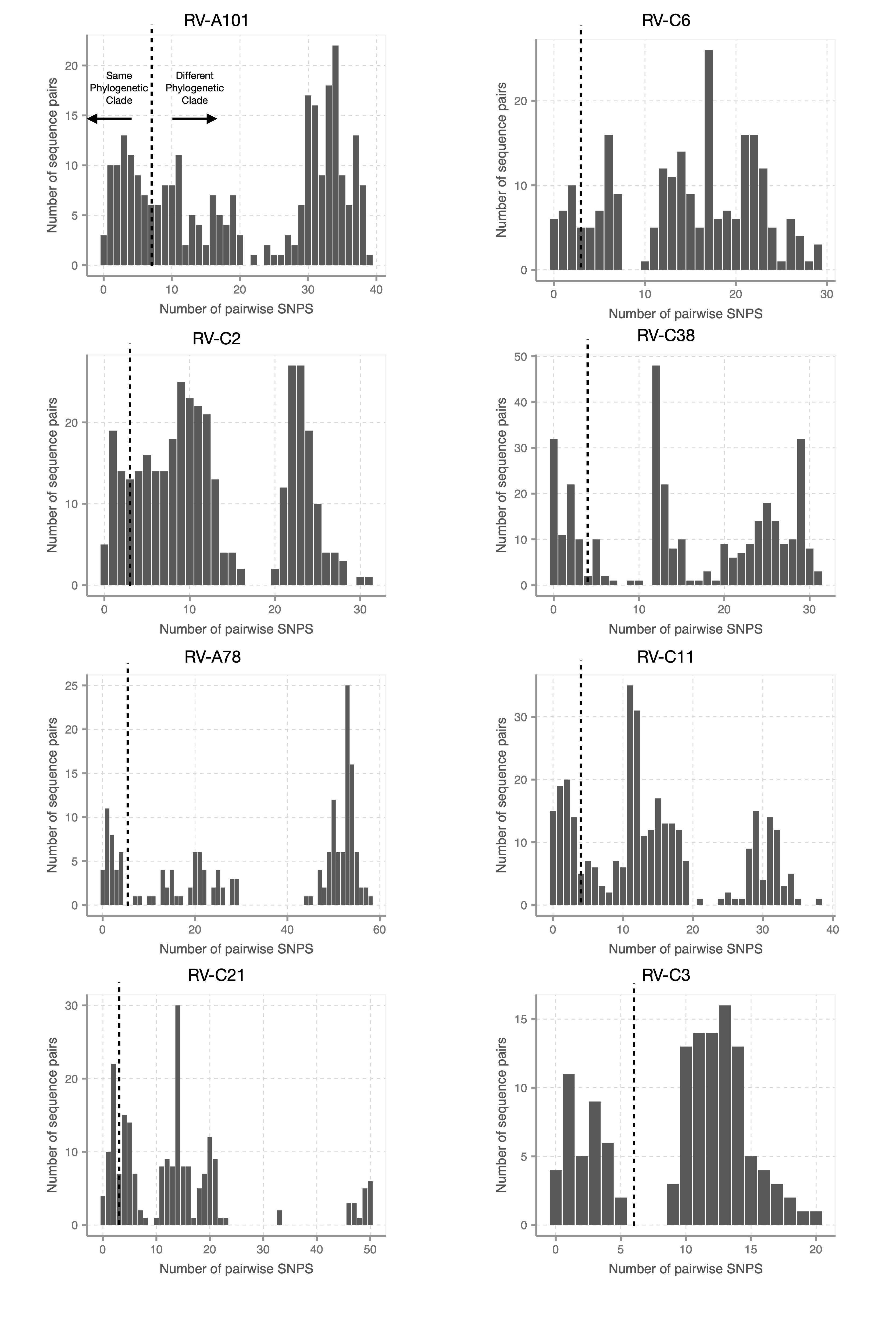

Supplement: ofab571_suppl_Supplementary_Figure_S2 [file ofab571_suppl_supplementary_figure_s2.jpeg]

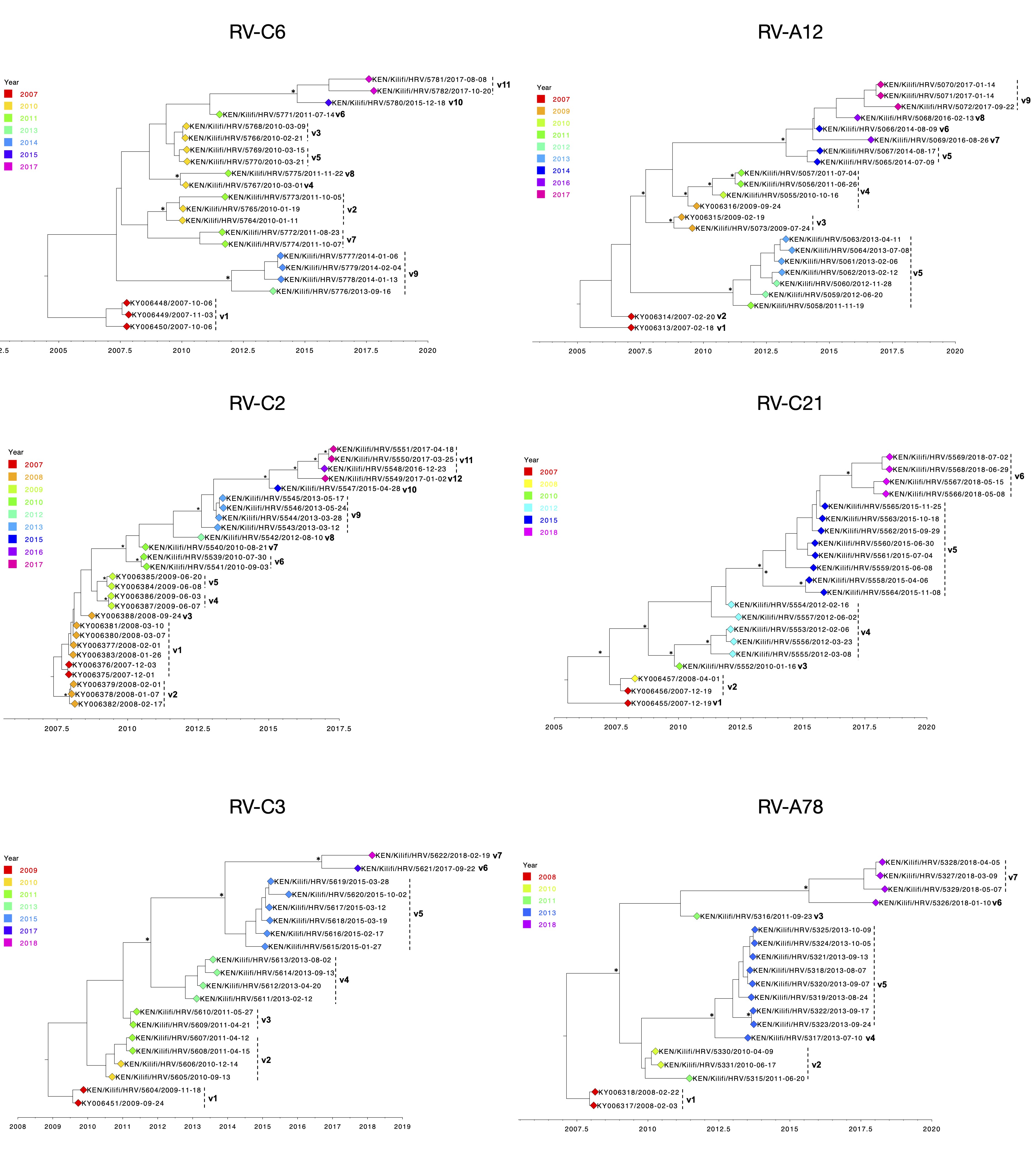

Supplement: ofab571_suppl_Supplementary_Figure_S3 [file ofab571_suppl_supplementary_figure_s3.jpeg]
