# Supplementary material for: Trends and Intensity of Rhinovirus Invasions in Kilifi, Coastal Kenya, Over a 12-Year Period, 2007–2018
Source: Open Forum Infect Dis. 2021 Nov 16;8(12):ofab571. doi: 10.1093/ofid/ofab571 (PMC8694214; doi:10.1093/ofid/ofab571)
Supplement: ofab571_suppl_Supplementary_Materials [file ofab571_suppl_supplementary_materials.docx]

**Supplementary Materials**

**Supplementary File 1** Monthly distribution and frequency of all the 144 RV types detected in Kilifi, Kenya, between 2007 and 2018.

**Supplementary Table 1** Summary of the number of samples tested, samples positive for RV, and sequences obtained from pediatric patients (< 60 months old) admitted at the Kilifi County Hospital with acute respiratory illness.

**Supplementary Table 2** Untyped VP4/VP2 sequences and p-distance to the closest RV reference sequence.

**Supplementary Figure 1** Nucleotide variability across the sequenced VP4/VP2 region for RV types A101, C6, C2, C38, A78, C11, C21 and C3. For each type, the viruses were compared to the earliest sampled sequence. Vertical coloured bars show the nucleotide differences: red is a change to T, orange is a change to A, purple is a change to C and blue is a change to G.

**Supplementary Figure 2** Distribution of pairwise nucleotide difference for the VP4/VP2 region of RV types A101, C6, C2, C38, A78, C11, C21 and C3.

**Supplementary Figure 3** Bayesian phylogenetic trees showing the VP4/VP2 region of the RV types A101, A12, C6, C21, C3 and A78. Variant names are next to the phylogenetic clusters, e.g., v1 representing variant 1 for the various types. Node support is indicated b (*) for posterior probabilities > 0.9.
